# Intravenous infusion of rocuronium bromide prolongs emergence from propofol anesthesia in rats

**DOI:** 10.1371/journal.pone.0246858

**Published:** 2021-02-11

**Authors:** Kaoru Suzuki, Hiroshi Sunaga, Kentaro Yamakawa, Yoshifumi Suga, Ichiro Kondo, Tsunehisa Tsubokawa, Shoichi Uezono

**Affiliations:** Department of Anesthesiology, The Jikei University School of Medicine, Tokyo, Japan; Massachusetts General Hospital, UNITED STATES

## Abstract

**Background:**

Neuromuscular blocking agents induce muscle paralysis via the prevention of synaptic transmission at the neuromuscular junction and may have additional effects at other sites of action. With regard to potential effects of neuromuscular blocking agents on the central nervous system, a definitive view has not been established. We investigated whether intravenous infusion of rocuronium bromide affects the emergence from propofol anesthesia.

**Methods:**

Using an in vivo rat model, we performed propofol infusion for 60 minutes, along with rocuronium bromide at various infusion rates or normal saline. Sugammadex or normal saline was injected at the end of the infusion period, and we evaluated the time to emergence from propofol anesthesia. We also examined the neuromuscular blocking, circulatory, and respiratory properties of propofol infusion along with rocuronium bromide infusion to ascertain possible factors affecting emergence.

**Results:**

Intravenous infusion of rocuronium bromide dose-dependently increased the time to emergence from propofol anesthesia. Sugammadex administered after propofol infusion not containing rocuronium bromide did not affect the time to emergence. Mean arterial pressure, heart rate, partial pressures of oxygen and carbon dioxide, and pH were not affected by rocuronium bromide infusion. Neuromuscular blockade induced by rocuronium bromide, even at the greatest infusion rate in the emergence experiment, was rapidly antagonized by sugammadex.

**Conclusions:**

These results suggest that intravenous infusion of rocuronium bromide dose-dependently delays the emergence from propofol anesthesia in rats. Future studies, such as detection of rocuronium in the cerebrospinal fluid or central nervous system, electrophysiologic studies, microinjection of sugammadex into the brain, etc., are necessary to determine the mechanism of this effect.

## Introduction

The major effect of neuromuscular blocking agents (NMBAs) is muscle paralysis induced via the prevention of synaptic transmission at the neuromuscular junction. In addition, NMBAs have potential effects at other sites of action. For example, rocuronium has been reported to have a vagal blocking effect at clinically relevant doses and a bronchoconstrictive effect at high doses by interacting with muscarinic receptors [1,2]. With regard to the effect of NMBAs on the central nervous system (CNS), a definitive view has not been determined [3–8]; however, a recent study reported that rapid and complete reversal of rocuronium-induced neuromuscular blockade with sugammadex lightened the depth of anesthesia [9]. The precise mechanism was not elucidated. Nonetheless, those results suggested that rocuronium might have an effect on the CNS. In the present study, we investigated whether intravenous infusion of rocuronium bromide affects the emergence from propofol anesthesia in an in vivo rat model of neuromuscular blockade and emergence. We also assessed the neuromuscular blocking, circulatory, and respiratory properties of rocuronium bromide infusion to examine possible factors affecting emergence.

## Materials and methods

Male Sprague Dawley rats (Japan SLC, Shizuoka, Japan) weighing 294 ± 10 g and aged 9 to 10 weeks were used (total *n* = 48 rats). Rats were housed with a 12-hour light-dark cycle with lights on at 7 am in a temperature- (22 ± 2°C) and humidity- (55 ± 10%) controlled room and allowed free access to water and food in a polymethylpentene cage with paper bedding (PaperClean; Japan SLC). All protocols were approved by the Institutional Animal Care and Use Committee of The Jikei University (approval number 2016–023). Animal care and experiments conformed to the Fundamental Guidelines for Proper Conduct of Animal Experiment and Related Activities in Academic Research Institutions by the Ministry of Education, Culture, Sports, Science and Technology of Japan. Surgery was performed under isoflurane anesthesia with lidocaine, and all efforts were made to minimize suffering.

### Reagents

Powdered rocuronium bromide was purchased from Tokyo Chemical Industry (Tokyo, Japan) and diluted in normal saline. Clinical formulations of propofol (Maruishi Pharmaceutical, Osaka, Japan) and sugammadex (MSD, Tokyo, Japan) were used.

### Experimental setup

Rats were anesthetized with isoflurane. After induction of anesthesia at 4% to 5%, isoflurane was maintained at 2% to 3%. The surgical procedure was initiated after confirming no response to foot pinch. Lidocaine was infiltrated into the incision sites. Respiratory rate and response to foot pinch were monitored during surgery, and the concentration of isoflurane was increased when an increase in respiratory rate or a withdrawal response to foot pinch was observed. The trachea was cannulated with a 16-g catheter, and the rat was ventilated at 10 mL/kg and 60 breaths/min with isoflurane and oxygen with a rodent ventilator (model 683; Harvard Apparatus, Holliston, MA, USA). A heating device was used to maintain body temperature at 36°C to 37°C. For the emergence, circulation, and respiration experiments, the right femoral vein was cannulated with a 24-g catheter for drug administration. For the neuromuscular blockade (NMB) experiment, the right jugular vein was cannulated for drug administration to keep the lower limb intact for neuromuscular monitoring.

### Assessment of the effect of rocuronium bromide on time to emergence

Rats were randomly allocated to the rocuronium- or normal saline-infusion group. After completion of the experimental setup, isoflurane was discontinued. When the concentration of isoflurane decreased to 1%, propofol infusion was initiated with a bolus dose of 15 mg/kg and continued at a rate of 40 mg/kg/h. For the rocuronium group (*n* = 18), rocuronium bromide was administered as an initial intravenous bolus of 5 mg/kg followed by continuous infusion at a rate of 250, 500, or 1000 μg/kg/min along with propofol infusion for 60 min. At the end of the infusion period, sugammadex (32 mg/kg) was injected, and the line was flushed with 0.5 mL normal saline. In a separate group of rats, normal saline was administered as a continuous infusion at a rate of 1.5 mL/kg/h (in the same volume as the rocuronium group) along with propofol infusion for 60 min. At the end of infusion, sugammadex (32 mg/kg; *n* = 6) or 0.1 mL normal saline (*n* = 6) was injected, and the line was flushed with 0.5 mL normal saline. In both groups, the vibrissae were pulled at intervals of 30 s until a sign of emergence (movement of tongue, mouth, or limbs) was observed. The time to emergence from propofol anesthesia, defined as the time from flush of the intravenous line at the termination of infusion to the appearance of a sign of emergence, was assessed. In general, the time to emergence should be defined as the time to return of the righting reflex [10,11]. However, because the return of the righting reflex appears after forelimb movement or mastication in experimental animals during emergence from anesthesia [12],  that definition should be inadequate for more invasive studies in which tracheotomy and mechanical ventilation are required. Hence, the time to emergence from propofol anesthesia was defined as the time from flush of the intravenous line at the termination of infusion to the appearance of movement of the tongue, mouth, or limbs.

### Assessment of neuromuscular blocking, circulatory, and respiratory properties of rocuronium bromide

After completion of the experimental setup, the neuromuscular blocking, circulatory, and respiratory properties of rocuronium bromide infusion were assessed in separate groups of rats. For the NMB experiment (*n* = 6), the sciatic nerve was exposed to attach a small slide electrode, and the tendon of the gastrocnemius muscle was connected to a force transducer (MLTF500/ST; ADInstruments, Colorado Springs, CO, USA). The sciatic nerve was stimulated at 0.1 Hz with a supramaximal stimulation of 0.2-ms pulse width using an electric stimulator (SEN-3401; Nihon Kohden, Tokyo, Japan) and an isolator (SS-104J; Nihon Kohden) to elicit twitch responses of the gastrocnemius muscle. Single twitch responses were amplified and recorded using PowerLab (ADInstruments). After establishment of the magnitude of the twitch responses, isoflurane was discontinued. When the concentration of isoflurane decreased to 1%, propofol infusion was initiated with a bolus dose of 15 mg/kg and continued at a rate of 40 mg/kg/h. Rocuronium bromide was administered as an initial intravenous bolus of 5 mg/kg followed by continuous infusion at a rate of 1000 μg/kg/min along with propofol infusion for 60 min. At the end of infusion, sugammadex (32 mg/kg) was injected, and the line was flushed with 0.5 mL normal saline. Both NMB and antagonism were assessed, and the time from injection of sugammadex to complete recovery of twitch height was determined.

For the assessment of circulatory and respiratory effects, rats were randomly allocated to the rocuronium- or normal saline-infusion group. The carotid artery was cannulated with a 24-g catheter and connected to a pressure transducer. Arterial pressure and heart rate (HR) were recorded throughout the experiment with PowerLab. Propofol infusion was performed in the same manner as that for the emergence experiment. Infusion of rocuronium bromide at 1000 μg/kg/min after an initial bolus of 5 mg/kg (rocuronium group; *n* = 6) or normal saline at 1.5 mL/kg/h (normal saline group; *n* = 6) was performed along with propofol infusion for 60 min. At the end of infusion, the line was flushed with 32-mg/kg sugammadex followed by 0.5 mL normal saline for the rocuronium group or 0.6 mL normal saline for the normal saline group. For blood gas analysis, 0.1 mL of blood was collected via the 24-g catheter inserted in the carotid artery at the following time points, when the monitoring of mean arterial pressure and heart rate was temporarily suspended: before infusion, at 30 min after initiation of infusion, and at 3 min after flushing the line at the discontinuation of infusion.

All experiments were conducted in a laboratory during the light phase (7 am to 7 pm). Each rat was studied once and killed by intravenous pentobarbital overdose after the experiment.

### Data analysis

The primary outcome was the relation between the time to emergence from propofol anesthesia and the infusion rate of rocuronium bromide along with propofol. The Spearman rank correlation coefficient (ρ) was used to analyze the relation between time to emergence and infusion rate of rocuronium bromide. We estimated that the correlation coefficient would be approximately 0.65, and to achieve a power of 80% and a type 1 error of 0.05, 16 samples were required. Statistical comparison was made by unpaired two-tailed Student *t* test to ascertain whether sugammadex alone was related to time to emergence from propofol anesthesia. Mean arterial pressure (MAP) and HR at bolus injection of propofol and rocuronium bromide (time 0) and at 1, 3, 5, 10, 20, 30, 45, and 60 min after initiating rocuronium bromide infusion with propofol, as well as the partial pressures of oxygen (PaO_2_) and carbon dioxide (PaCO_2_) and pH before infusion, at 30 min after initiating infusion, and at 3 min after flushing the line at the end of infusion, were compared to those in response to normal saline administration with propofol using two-way repeated measures analysis of variance with a post hoc Student *t* test and Bonferroni correction for multiple comparisons. SigmaPlot 13 (Systat Software, San Jose, CA, USA) was used for analysis. All values are presented as mean ± SD. For all statistical comparisons, a value of *p* < 0.05 was considered significant.

## Results

**Fig 1:**
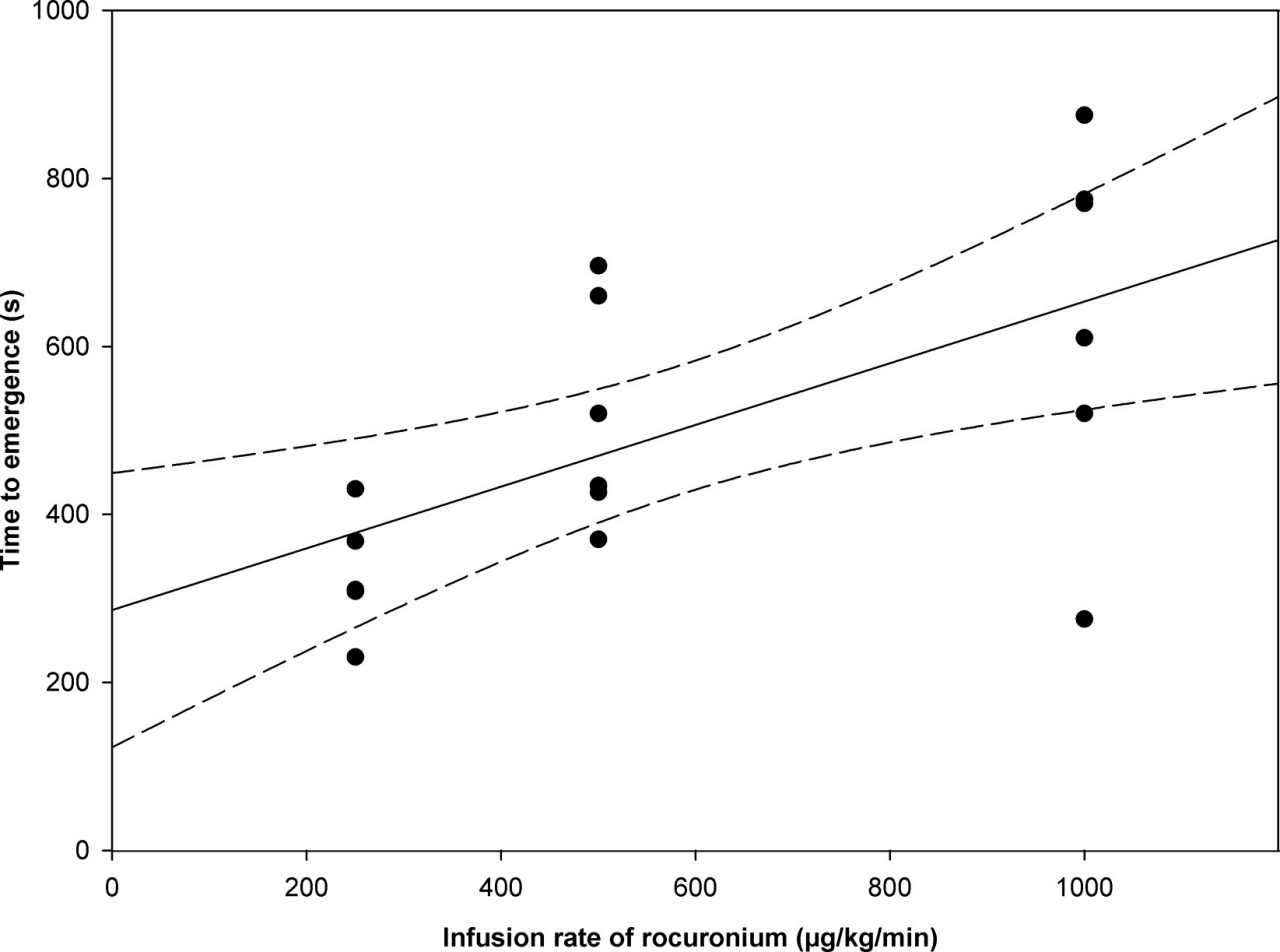
Relation between time to emergence from propofol anesthesia and infusion rate of rocuronium bromide along with propofol using a linear regression model (solid line) with corresponding 95% CIs (dashed lines). Individual responses at each dose (250, 500, 1000 μg/kg/min) are shown as black circles. The time to emergence from propofol anesthesia was dose-dependently prolonged by simultaneous infusion of rocuronium bromide (ρ = 0.624, *p* = 0.006).

The time to emergence from propofol anesthesia was 239 ± 94 s after simultaneous infusion of normal saline without rocuronium bromide. When rocuronium bromide was administered at a rate of 250, 500, and 1000 μg/kg/min along with propofol, the time to emergence was 346 ± 78, 518 ± 134, and 638 ± 219 s, respectively. The relation between the time to emergence from propofol anesthesia and the infusion rate of rocuronium bromide is shown in Fig 1. The Spearman rank correlation coefficient suggested that the time to emergence from propofol anesthesia was dose-dependently prolonged by simultaneous infusion of rocuronium bromide (ρ = 0.624; *p* = 0.006). Sugammadex alone did not affect the time to emergence from propofol anesthesia (280 ± 60 s; *p* = 0.39). Muscle twitch was completely blocked throughout rocuronium bromide infusion at a rate of 1000 μg/kg/min in all rats and was completely recovered at 99 ± 21 s by 32 mg/kg sugammadex.

**Fig 2:**
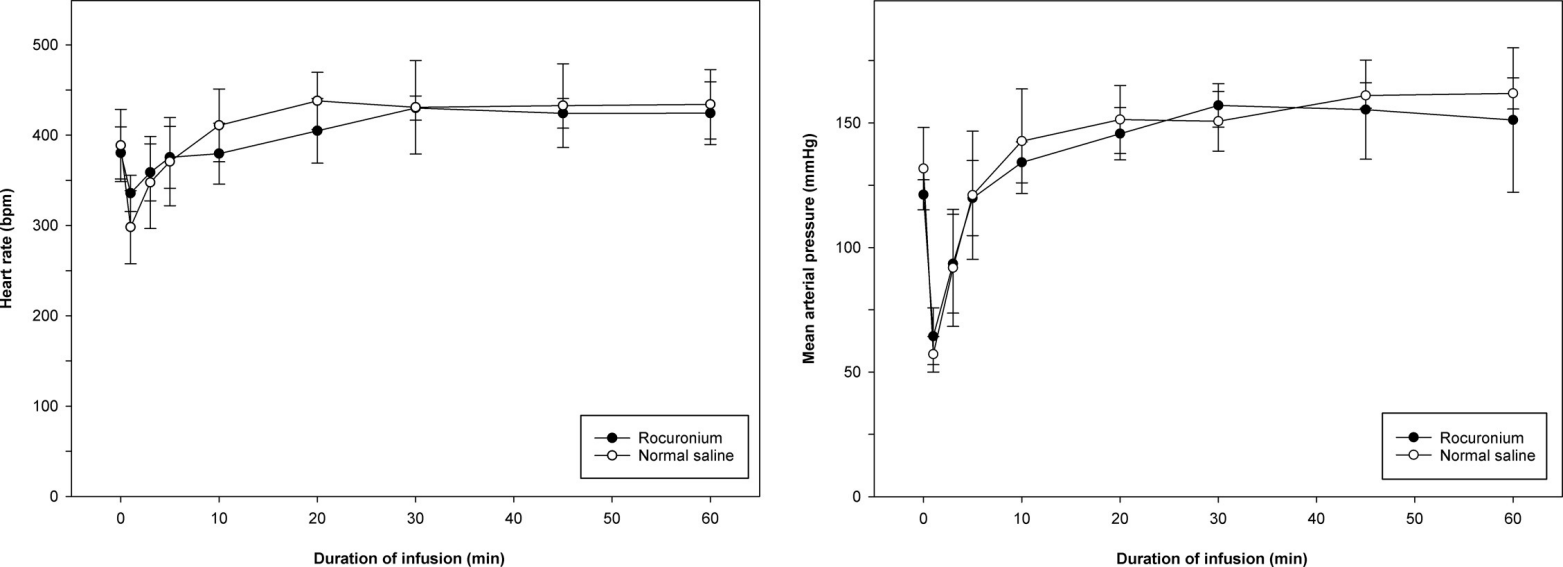
Mean arterial pressure (mmHg) and heart rate (beats per minute [bpm]) measured at bolus injection of propofol (time 0) and at 1, 3, 5, 10, 20, 30, 45, and 60 min after initiating propofol infusion with rocuronium or normal saline. (A) Mean arterial pressure after initiating propofol infusion with rocuronium did not differ significantly from that with normal saline at any time point. (B) Heart rate after initiating propofol infusion with rocuronium did not differ significantly from that with normal saline at any time point.

Values for MAP and HR during rocuronium bromide or normal saline infusion with propofol are shown in Fig 2. Values for MAP and HR showed significant differences between time points in each group; however, those during rocuronium bromide infusion did not differ significantly from those during normal saline infusion at any time point.

**Fig 3:**
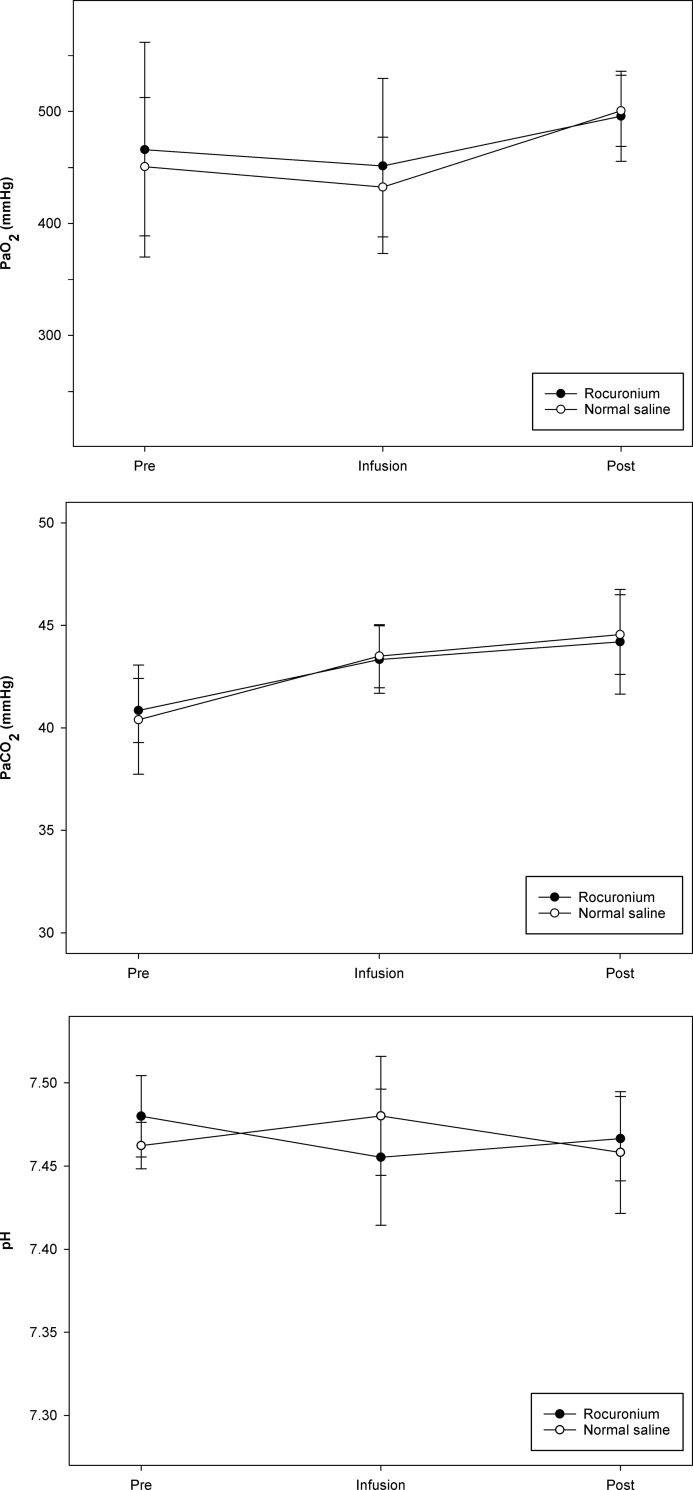
Blood gas analysis was performed at the following time points: Before infusion (Pre), at 30 min after initiation of infusion (Infusion), and at 3 min after flushing the line at the discontinuation of infusion (Post). (A) Partial pressure of oxygen (PaO_2_). (B) Partial pressure of carbon dioxide (PaCO_2_). (C) pH. Values for PaO_2,_ PaCO_2_, and pH did not differ significantly between the rocuronium group and the normal saline group at any time point.

Values for PaO_2,_ PaCO_2_, and pH before infusion, at 30 min after initiating infusion, and at 3 min after flushing the line at the end of infusion are shown in Fig 3. Values for PaO_2_ showed a significant difference between time points in the normal saline group, and those for PaCO_2_ showed significant differences between time points in each group; however, values for PaO_2,_ PaCO_2_, and pH did not differ significantly between the rocuronium group and the normal saline group at any time point.

## Discussion

The results of the present study show that continuous intravenous infusion of rocuronium bromide dose-dependently delayed the emergence from propofol anesthesia in rats. The definitive mechanism was not elucidated; however, some possible factors affecting emergence could be eliminated. Sugammadex rapidly reversed rocuronium-induced NMB; therefore, the rats were not immobilized despite potentially being aware. In addition, sugammadex alone did not affect the time to emergence. The effect of propofol infusion with rocuronium bromide on circulation and respiration did not differ from propofol infusion without rocuronium bromide; therefore, low cerebral blood flow or slow metabolism of propofol was not a contributing factor.

The action of NMBAs is to induce muscle paralysis owing to the prevention of neuromuscular transmission at the neuromuscular junction, and there may be effects at other sites of action [1,2]. Concerning the effect of NMBAs on the CNS, a conclusive view has not been established. Some studies have reported effects on the CNS [3,5,6], whereas others have reported no effect [4,7,8]. One of the reasons for this discrepancy might be evaluation method. Because electromyography activity can contaminate electroencephalography signals, it might be difficult to assess the effect of NMBAs on the CNS with electroencephalography or bispectral index monitoring. A recent study evaluated clinical signs and reported that the reversal of neuromuscular blockade with sugammadex might be associated with awakening; that is, rocuronium potentially had some effect on the CNS [9]. The present study provides another relevant finding that continuous intravenous infusion of rocuronium bromide has a dose-dependent effect of delaying the emergence from propofol anesthesia.

The mechanism underlying the effect of intravenous infusion of rocuronium bromide on emergence from propofol anesthesia is unclear. One possible mechanism for the rocuronium-induced delay in rats might be a direct effect on the CNS by entering the cerebrum. In general, NMBAs pass the blood-brain barrier (BBB) minimally because they are large quaternary ammonium compounds. However, it has been reported that the immature BBB in neonates or disrupted BBB in patients with certain clinical conditions might have increased permeability, resulting in the passage of rocuronium from blood vessels to the cerebrum [13–16]. In addition, the permeability of rocuronium might differ between rodents and primates because of species-specific differences in the BBB [17]; rocuronium might penetrate more easily in rats. Furthermore, the dosage for continuous infusion of NMBAs required to maintain a consistent level of NMB varies among species. Whereas the infusion rate of rocuronium to obtain twitch depression of 90% to 95% (90–95% effective dose [ED_90–95_]) in humans is reported to be 9 μg/kg/min, the ED_90_ for NMB with continuous infusion of rocuronium in rats is 119 μg/kg/min [18,19]. The infusion rates in the present study, 250, 500, and 1000 μg/kg/min, were equal to approximately 2, 4, and 8 times the ED_90_ for NMB, respectively; therefore, they might not be excessive doses for this animal model. Nevertheless, access to the cerebrum might have been facilitated by the large number of molecules administered during infusion.

It is unclear what the effects of NMBAs administered via intravenous infusion might be if they should pass across the BBB. The effects of NMBAs administered directly to the cerebrospinal fluid or brain slices have been reported [20–23], and the results imply that NMBAs act at several locations in the cerebrum and have a variety of effects. We previously reported that microinjection of pancuronium into the lateral ventricle at doses of 1.6% to 16% of the ED_50_ for NMB in rats dose-dependently enhanced the depth of isoflurane anesthesia [20]. Another study reported pancuronium-induced seizures when a dose of 2.3 times the ED_50_ for NMB was injected into the lateral ventricle in rats [21]. These findings suggest that pancuronium might have an excitatory effect at high concentrations and an inhibitory effect at low concentrations in the area of the lateral ventricle. Rocuronium might have similar effects because it is an analog of pancuronium. If seizures had occurred in the present study, the rats could not move because of complete NMB during intravenous infusion. The concentration of rocuronium in the cerebrospinal fluid was not measured in the present study. Thus, it is difficult to speculate as to whether rocuronium showed an excitatory or inhibitory effect on the cerebrum. However, because either effect could result in delayed emergence from general anesthesia, a direct action of rocuronium on the CNS could be considered a probable reason.

Another possible mechanism might be an indirect effect of rocuronium on the CNS. One study suggested indirect inhibition of cerebral stimulation by pancuronium as a result of a decrease in muscle afferent activity [24]. A decrease of afferent input to the CNS could enhance the depth of anesthesia. The effect might be present during rocuronium infusion in the present study; however, after recovery from NMB, it might not. Sugammadex completely recovered the twitch responses of the gastrocnemius muscle at 99 ± 21 s after the discontinuation of rocuronium infusion at a rate of 1000 μg/kg/min. Because the muscles of the limbs are more sensitive to the effects of NMBAs than other muscles, which explains the slower recovery from NMB [25], the muscle tone of almost all skeletal muscles should have been recovered from NMB at the time of complete reversal of NMB of the gastrocnemius muscle. Hence, the decrease in muscle afferent activity should have been wearing off at the same time. An indirect action on the CNS would not be expected to affect the time to emergence in the present study. Furthermore, considering the time to emergence of 638 ± 219 s, it is unlikely that the rats were immobilized despite potentially being aware.

Sugammadex has a molar mass of 2178 g/mol, which is approximately 3.5 times greater than the molar mass of rocuronium. Hence, sugammadex generally binds rocuronium in the blood vessels and cannot move outside the blood vessels. According to the concentration gradient, rocuronium moves to the blood vessels from the distributed tissue. Sugammadex should have removed rocuronium from the CNS in the present study. Nevertheless, because the CNS has greater sensitivity to NMBAs [20], even a small number of rocuronium molecules remaining in the CNS after intravenous injection of sugammadex might have acted to slow the recovery of awareness during emergence.

Circulatory effects might cause delayed emergence from propofol anesthesia because they might alter cerebral blood flow as well as the metabolism of propofol, which influences the plasma concentration [26]. The PaCO_2_ and pH might affect cerebral blood flow as well. Compared with normal saline infusion with propofol, rocuronium infusion with propofol did not elicit significant differences in MAP, HR, PaCO_2_, PaO_2_, or pH at any time point in the present study; therefore, circulatory and respiratory effects on the emergence from propofol anesthesia should be excluded.

The present study was conducted with rocuronium bromide. Bromide is used as an anticonvulsant for animals with epilepsy and potentially has a sedative effect [27]. A decrease of response rate, sluggishness, or sleepiness is reported to occur at an intraperitoneal dose of 300 mg/kg of bromide ion in rats [28]. The greatest cumulative dose of bromide ion administered as rocuronium bromide in the present study was approximately 8.45 mg/kg. This large difference in dose of bromide ion implies that the dose administered in the present study was not large enough to affect the CNS even if the difference of administration routes was considered. Nevertheless, to verify that bromide ion has no impact on the emergence from propofol anesthesia, a comparison with sodium bromide or potassium bromide infusion is necessary.

A limitation of this study is that we did not elucidate the definitive mechanism underlying the delayed emergence from propofol anesthesia with intravenous infusion of rocuronium bromide. Studies to detect rocuronium in the cerebrospinal fluid or CNS, as well as electrophysiologic studies, would be necessary to obtain supportive evidence. Furthermore, microinjection of sugammadex into the cerebrum might provide determinative findings. Even if the microinjection of sugammadex does not antagonize the delayed emergence, it may not be able to negate the possibility of the direct effect of rocuronium on the CNS because the specific location at which rocuronium acts in the cerebrum has not been identified and sugammadex could induce neuronal damage [29,30]. However, if the microinjection is associated with the antagonism of the delayed emergence, it would prove that the effect was induced by rocuronium which passed across the BBB. Further study will be warranted to elucidate the mechanism underlying the observed effect.

## Conclusions

Our present results show that continuous intravenous infusion of rocuronium bromide dose-dependently delayed the emergence from propofol anesthesia in rats. This is not inconsistent with the signs described in case reports and a previous study that indicated CNS effects of NMBAs [9,13,14]. Future studies, such as detection of rocuronium in the cerebrospinal fluid or CNS, electrophysiologic studies, microinjection of sugammadex into the brain, etc., are necessary to obtain conclusive evidence regarding the mechanism underlying this effect.

## Supporting information

S1 File Database with raw data for the emergence experiment. (XLSX)

S2 File Database with raw data for the neuromuscular blockade experiment. (XLSX)

S3 File Database with raw data for the circulation experiment. (XLSX)

S4 File Database with raw data for the respiration experiment. (XLSX)
